# Aβ Deposition in Extracellular Space Disrupts Glial-Neuron Communication and Triggers Alzheimer's Disease

**DOI:** 10.14336/AD.2025.0873

**Published:** 2026-10-04

**Authors:** Ying Chen, Ling Ding, Ziqi Gu, Zixi Tian, Tingting Gao, Feiyan Zhou, Shouzi Zhang, Zhiqian Tong

**Affiliations:** ^1^Beijing Geriatric Hospital, Beijing, 10095, China.; ^2^Zhejiang Provincial Clinical Research Center for Mental Disorders, The Affiliated Wenzhou Kangning Hospital, School of Mental Health, Wenzhou Medical University, Wenzhou, 300025, China.; ^3^Peking University Sixth Hospital, Peking University Institute of Mental Health, NHC Key Laboratory of Mental Health (Peking University), National Clinical Research Center for Mental Disorders (Peking University Sixth Hospital), Beijing 100191, China.; ^4^College of Mental Health, Inner Mongolia Medical University, 010059, China.

**Keywords:** Alzheimer's Disease, Cell communication, Extracellular space (ECS), Formaldehyde (FA), Interstitial fluid (ISF)

## Abstract

Disruptions in glial-neuron communication are linked to brain diseases; however, the exact mechanisms are not yet fully understood. This review highlights the critical role of glia-neuron interactions in sustaining normal brain functions. Under physiological conditions, astrocytes, microglia and oligodendrocytes, collaboratively regulate the homeostasis of brain structures and functions through diverse communication mechanisms. Conversely, under pathological conditions, disorders in glial-neuron communication can precipitate in neurodegenerative diseases, such as Alzheimer's Disease (AD). In AD, astrocytes use APOE to increase amyloid-beta (Aβ) deposition in the brain extracellular space (ECS) and neuron death, while microglia overactivation causes Tau overexpression and oligodendrocyte demyelination, leading to communication issues. Toxic proteins, Aβ and Tau, block brain ECS and hinder the drainage of interstitial fluid (ISF) cause the vicious cycle. The obstruction of ISF drainage certainly impedes neurotransmitter transmission, nutrient delivery, and waste removal, ultimately leading to neuronal death and cognitive decline in AD, which is also a direct factor contributing to the failure of drug delivery. Age-associated formaldehyde (FA) may act as a detrimental factor that exacerbates Aβ aggregation and promotes tau hyperphosphorylation, further aggravating ECS and ISF dysfunction. Interestingly, the interrupting the pathological cycle of FA-promoted Aβ aggregation and Aβ-induced FA generation by phototherapy and nanomedicine has been found to restore the ECS architecture and ISF drainage, which effectively improves AD symptoms. Hence, the re-establishing ECS structure and communication between neurons and glial cells may offer a promising therapeutic strategy for treating AD.

## 1. Introduction

Glial cells are extensively distributed in the brains of mammals, and early studies suggested that glial cells primarily serve a supportive role, which is why they were named 'glia' (from the Greek word for glue) [1]. With the advancement of neuroscience and magnetic resonance imaging technology, scientists have observed the dynamic changes and functional processes of glial cells in the brains of living animals, which has advanced research on the complex neural networks formed by neurons and glial cells [2]. Thus, according to recent research advancements, scientists have gradually realized that, in addition to their supportive role, glial cells also function in maintaining microenvironmental homeostasis, participating in information transmission, and clearing metabolic waste [3]. Recent research on neurodegenerative diseases is increasingly focusing on glial cells due to their widespread presence and varied roles. This review highlights the communication between neurons and glial cells like astrocytes, microglia, and oligodendrocytes, examines how communication issues contribute to Alzheimer's Disease (AD), and suggests new treatment strategies for AD.

## 2. Classification and Functions of Glial Cells

### 2.1 Astrocytes

Astrocytes have a diameter of 3-5 μm and are the largest type of glial cells. They are star-shaped, with multiple branches extending from the cell body. These branches progressively divide, intricately intertwining with thousands of synapses, blood vessels, and other cells, forming a grand and spectacular structure [4]. Based on their distribution and morphological characteristics, astrocytes can be roughly divided into protoplasmic astrocytes and fibrous astrocytes [1]. Protoplasmic astrocytes are typically located in the gray matter of the central nervous system and are relatively thick and short; fibrous astrocytes are predominantly found in the white matter and are relatively thin and long [5]. Protoplasmic astrocytes envelop synapses and blood vessels, while fibrous astrocytes connect with nodes of Ranvier. Both types form gap junctions with nearby astrocytes to supply energy metabolites to neurons in the gray and white matter [6].

Mature astrocytes play a significant role in maintaining homeostasis in the central nervous system. Fluorescent phagocytosis reports indicate that astrocytes can engulf redundant synapses in the CA1 region of the hippocampus in adult mice to maintain circuit homeostasis [7]. Besides mouse neurons, astrocytes can also regulate synaptic functions between human neurons [8]. A previous study demonstrated that astrocytes in the optic nerve head can continuously phagocytose the axonal organelles of undamaged optic nerves through Mac-2 (a bridging molecule that mediates the binding of target fragments to phagocytic receptors) [9-11]. Thus, astrocytes contribute to synaptic regulation and circuit stability under physiological conditions. In pathological conditions such as AD, however, their clearance and homeostatic roles may become impaired, leading to reactive gliosis and disruption of glymphatic clearance (see Section 4).

### 2.2 Microglia

The close association between the immune system and mental disorders was proposed by F. C. Bennett et al. in 2019, elucidating the impact of the active immune system in the brain on AD from a basic scientific perspective [12]. Around embryonic days 13.5-14.5, early microglia develop into pre-microglia, and in the weeks following birth, pre-microglia mature into adult microglia, with each stage of microglia having distinct functions [13]. As the resident immune cells in the brain, microglia primarily play a surveillance role: they control the number of neurons and synapses during neural system development, maintain the brain microenvironment's homeostasis, and consolidate neural circuits [14, 15]. Research on the development of the mouse brain has shown that microglia can regulate the number of neurons by actively inducing apoptosis and removing excess, apoptotic, or dead neural progenitor cells (NPCs) [16]. Similarly, during the development of the human central nervous system, microglia support the survival, proliferation, and maturation of neuronal progenitor cells and neurons, stimulate neurogenesis, and shape efficient neuronal circuits [17]. Moreover, microglia play a continuous role in pruning and remodeling dendritic spines [18]. Overall, microglia act as key regulators of neuronal number and synaptic remodeling in healthy brains. In disease contexts such as AD, their activation states change profoundly, with distinct consequences for neuroprotection or neurotoxicity (see Section 4.4).

### 2.3 Oligodendrocytes

Oligodendrocytes are derived from NG glial cells (oligodendrocyte precursor cells, OPCs), and are the most abundant cells in the brain's white matter, interspersed among myelinated nerve fibers [19-21]. Oligodendrocytes produce a lipid-rich membrane known as myelin, which wraps around axons to form the myelin sheath, supporting neurons and increasing the speed of neural action potential conduction [1]. The myelin sheath is not a permanent or static structure. With the occurrence of learning and cognitive events throughout life, the formation and degradation of myelin create a dynamic range known as myelin plasticity [22]. While encoding and storing information, oligodendrocyte precursor cells multiply and mature into oligodendrocytes, which form new myelin sheaths along axons, adjusting the length and thickness of existing sheaths [22-24]. In adult mice, learning new motor tasks helps generate new oligodendrocytes, and a higher rate of myelin formation is closely associated with improved learning and memory abilities [25, 26]. These findings indicate that oligodendrocytes and their myelin remodeling support neural plasticity in physiological conditions. In AD, however, this process becomes disrupted, leading to myelin loss and cognitive impairment (see Section 4.5) (Fig. 1).

**Figure 1.**
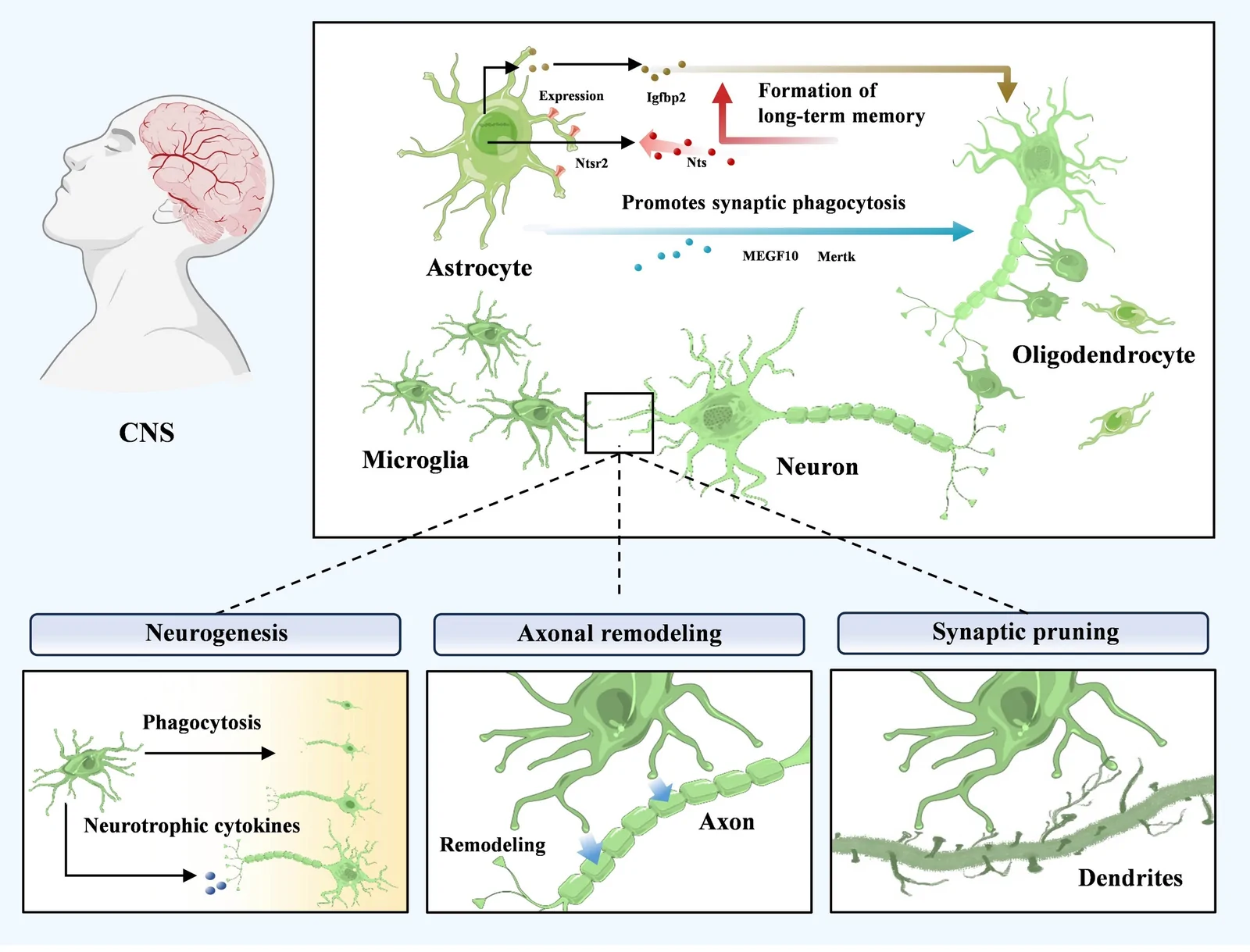
**Classification and function of glial cells under physiological conditions**. In the central nervous system, astrocytes play an important role in maintaining homeostasis, participate in the formation of memory, and mediate the formation of synapses. Microglia participate in Neurogenesis, Axonal remodeling, and Synaptic pruning through cytokines. Oligodendrocytes can form myelin to maintain the stability of the nervous system. Abbreviation: CNS: Central Nervous System.

## 3. Normal communications in CNS

### 3.1 Astrocytes-Microglia Crosstalk

The nuclear localization IL-33 reporter gene can detect astrocyte-specific expression of IL-33 in the spinal cord and thalamus. Under physiological conditions, IL-33 signals to microglia, promoting their phagocytosis of synapses and the development of neural circuits [27]. Astrocytes can also secrete IL-3, and microglia express the IL-3 receptor IL-3Rα. IL-3 acts on IL-3Rα, reducing the levels of soluble Aβ in microglia and inhibiting amyloid plaque formation [28]. Activated microglia induce reactive astrocytes by secreting Il-1α, TNF, and C1q. Reactive astrocytes further induce neuronal death and reduce the clearance of redundant synapses [29].

### 3.2 Astrocyte-Neuron Signaling

As the most abundant amino acid in the brain, glutamate is the primary excitatory neurotransmitter, involved in a range of physiological functions such as brain development, learning, memory, and cognition through synaptic action [30]. Glutamate receptors on astrocytes can be divided into metabotropic receptors (mGluRs) and ionotropic receptors (iGluRs) [31]. Metabotropic receptors can be subdivided into NMDA receptors (N-methyl-D-aspartate receptors, NMDAR), AMPA receptors (α-amino-3-hydroxy-5-methyl-4-isoxazole-propionic acid receptors, AMPAR), and KA receptors (Kainate receptors, KAR) [32]. Ionotropic receptors can be divided into three major groups (Group I, II, III), which are activated through G protein-coupled mechanisms. They do not directly control ion channels but regulate cellular functions by activating second messenger systems [33]. The NMDA receptor is assembled from two GluN1 subunits and two variable GluN2 subunits (including types 2A to 2D), and it is a crucial excitatory glutamate-regulated ion channel in the brain, playing a central role in mediating synaptic transmission and plasticity formation [34]. Glutamate released into the synaptic cleft can bind to NMDA receptors on the postsynaptic neuron and open the channel of the NMDA receptor, allowing calcium ions to enter the cell and induce changes in the neuronal internal potential [35]. A series of biochemical reactions occur within neurons, enhancing or weakening synaptic connection strength, thereby affecting the formation and consolidation of memory [36]. However, if glutamate is released excessively, it can cause sustained activation of NMDA receptors, leading to calcium overload in neurons and subsequently causing cell death [37].

In astrocytes, there are two efficient glutamate transporters, EAAT1 and EAAT2, which actively uptake extracellular glutamate, playing an indispensable and crucial role in maintaining glutamate homeostasis, regulating synaptic plasticity, and ensuring neuron survival [38]. When glutamate is released from the presynaptic neuron into the synaptic cleft, most of it is absorbed by astrocytes through EAAT2. Astrocytes utilize glutamine synthetase to convert glutamate into glutamine, which is then transported to the presynaptic neuron via the glutamine transporter [39]. Inside the neuron, phosphate-activated glutaminase further catalyzes the conversion of glutamine back into glutamate, ensuring that glutamate can once again serve as a neurotransmitter in synaptic transmission. Under the action of glutamate vesicular transporters (VGLUTs), glutamate enters the synaptic vesicles, and is subsequently released into the synaptic cleft as the vesicles release [40]. The importance of this cycle lies in its ability to efficiently recycle and reuse glutamate in the brain, which not only maintains stable neurotransmitter levels but also ensures the normal functioning of neurons.

In 2021, Moritz Armbruster and colleagues identified a previously unrecognized mode of astrocyte-neuron communication mediated by peripheral astrocytic processes (PAPs), which were shown to be pivotal regulators of synaptic plasticity [41, 42]. During neuronal activity, the depolarization of PAPs can inhibit astrocytes from clearing glutamate, thereby enhancing the activation effect of glutamate on neurons [41]. There is a large amount of excitatory amino acid transporters (EAATs) in PAPs, such as GLT1 and GLAST [43], It clears extracellular glutamate and regulates excitatory neuron transmission. Inward-rectifying potassium channels buffer potassium ions inside and outside the cell. During neuronal activity, increased extracellular potassium causes PAPs to depolarize, inhibiting EAATs and enhancing synaptic NMDA currents, thereby influencing synaptic plasticity [44]. The connections between astrocytes and synapses can be categorized into three types: a single synapse with an astrocyte branch, a single or multiple lobules of an astrocyte, or a single astrocyte branch or lobule connecting with multiple synapses. The dynamic changes in PAPs regulate neuronal plasticity by affecting neurotransmitter clearance, K^+^ dynamics, and the supply of glutamine [45].

Previous study has found that the formation of long-term memory requires the mutual communication between astrocytes and neurons [46]. Researchers used advanced transcriptomics to map memory trace spatial maps and identified 'imprinting astrocytes' that interact with P+T- neurons (with high Penk and low Tac expression) to form long-term memory. These astrocytes undergo lasting gene expression changes, with the neurotensin receptor gene Ntsr2 uniquely expressed in them. Neurons secrete neurotensin (Nts), which activates astrocyte receptors to release Igfbp2, aiding the transition from short-term to long-term memory [47, 48].

Furthermore, the communication between astrocytes and neurons may also occur through synaptic pruning and phagocytosis. Researchers have revealed that astrocytes lacking MEGF10 and MERTK exhibit lower phagocytic ability than wild-type astrocytes. This suggests that MEGF10 and Mertk are crucial for astrocytes to phagocytose neuronal synapses [49] (Fig. 2A).

### 3.3 Microglia-Synapse Interactions

In their resting state, microglia have a stable cell body with many fine branched protrusions, aiding in the real-time monitoring of the state and quantity of neurons and synapses [50]. Under normal physiological conditions of the central nervous system, microglia-neuron communication primarily manifests in three areas: neurogenesis, synaptic pruning, and axon remodeling. These functions collectively ensure the smooth operation of the CNS [51].

During neurogenesis, the communication between microglia and neurons can be divided into three stages: In the early stage, microglia release neurotrophic factors such as BDNF and NGF to promote the formation of synapses and connections between neurons; Pre-microglia release neurotrophic factors or cytokines to promote or inhibit the development of specific types of synapses; In adult microglia, excess synaptic connections or axons are removed through phagocytosis [13, 52]. This process is vital for refining and stabilizing neural networks. Researchers used light-sheet fluorescence microscopy and correlative light and electron microscopy (CLEM) to observe microglia interacting with synapses in developing hippocampal cultures. They found that microglia selectively perform partial phagocytosis, or 'trogocytosis', to prune presynaptic structures and trigger filopodia-like protrusions in postsynaptic dendritic spines [53]. Besides synapses, during the initial stages, neurons form numerous axons. Microglia directly contact and remove these excess axons, maintaining the stability of the neural circuit [54]. The Axon Initial Segment (AISM) is a specialized membrane region in the axon responsible for initiating action potentials [55]. In the cerebral cortex, a small number of microglia extend a single protrusion associated with and overlapping the AIS, participating in synaptic modification and maintenance of the neural circuit, ensuring normal communication functions between neurons [56] (Fig. 2B).

**Figure. 2:**
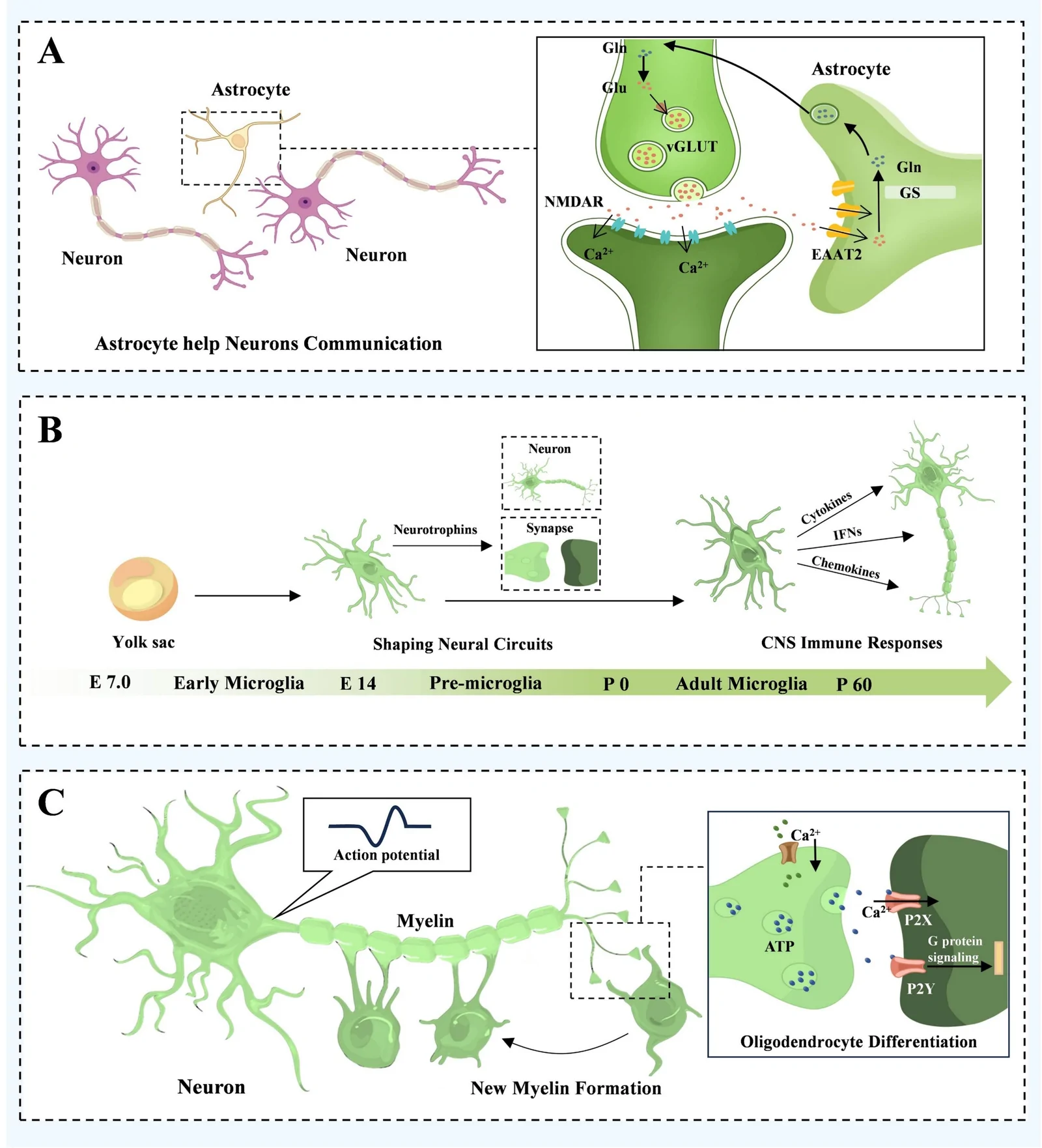
**Normal communications between glial cells and neurons**. (**A**) Astrocytes support neurons by recycling glutamate through high-affinity transporters EAAT1 and EAAT2, maintaining the glutamate cycle. (**B**) In early development, microglia promote synapse formation via neurotrophic factors such as BDNF and NGF. In adulthood, they secrete cytokines and interferons as key mediators of CNS immune responses. (**C**) Action potentials trigger Ca²^+^ influx and ATP release, activating P2X/P2Y receptors on OPCs. This promotes OPC differentiation into oligodendrocytes, which form myelin to support neuronal signal transmission. Abbreviations: Glu: Glutamate; vGLUT: Vesicular Glutamate Transporter; GS: Glutamine Synthetase; BDNF: Brain-Derived Neurotrophic Factor; NGF: Nerve Growth Factor.

Phi T. Nguyen et al. proposed that microglia-neuron communication can be achieved through the regulation of synaptic remodeling by IL-33. Hippocampal neurons can express IL-33b (a cytokine of the IL-1 family), which acts on IL-33 receptors on microglia, driving microglia to phagocytose ECM and clear chondroitin sulfate proteoglycan (CSPG) aggregates in the brain's extracellular space [57, 58]. CSPG is composed of a core protein covalently linked to chondroitin sulfate (CS) chains and is one of the main components of the ECM. As a barrier molecule, it can affect synaptic plasticity and the stability of the brain microenvironment [59]. The ECM is involved in the formation of Aβ plaques, promoting protein accumulation and fibrosis [60]. Under normal conditions, microglia actively communicate with neurons to clear CSPG, which can reduce protein accumulation in the ECM.

### 3.4 Oligodendrocyte-Axon Support

Although the process of myelin formation is controlled by intrinsic genetic programs and does not require neuronal activity, communication between neurons and OPCs is an important factor that can regulate the plasticity of the myelin sheath [61]. As a source of oligodendrocytes, oligodendrocyte precursor cells are abundantly present from birth and into adulthood, and have been shown to communicate with neurons through direct synaptic signals, although the specific mechanisms and significance remain unclear [1]. It is known that communication between neurons and OPCs mainly relies on glutamatergic and GABAergic synapses [62]. Glutamatergic neuron-OPCs synaptic communication influences oligodendrocyte proliferation and myelin sheath formation [63].

Glutamate is the principal excitatory neurotransmitter in the brain. Upon arrival of an action potential at the presynaptic terminal, depolarization opens voltage-gated Ca²^+^ channels, triggering vesicular release of glutamate into the synaptic cleft. The transmitter then activates ionotropic or metabotropic receptors, the latter being key modulators of neuron-oligodendrocyte precursor cell (OPC) synaptic plasticity [64-66]. There is currently no consensus on the role of ionotropic receptors in OPCs. Some studies suggest that activating ionotropic receptors, such as NMDARs, may regulate the developmental maturation process of OPCs. However, other studies indicate that activating ionotropic receptors, like NMDARs, has no effect on the function of OPCs [66-68]. For the specific role of metabotropic receptors in oligodendrocytes, very little is known [66]. Literature suggests that when metabotropic receptors are activated by specific agonists, they can promote the differentiation of OPCs in vitro [69]. Therefore, it can be said that neuron-OPC communication dependent on synaptic signals may be a fundamental mechanism for dynamically regulating the functions of neural circuits [70] (Fig. 2C).

## 4. AD-Related Alterations of Glia

### 4.1 Astrocytes in AD

#### 4.1.1 Reactive Gliosis in the Early Stages of AD

In the early stages of AD, astrocytes undergo reactive gliosis, characterized by cell swelling and hypertrophy, cell proliferation, and changes in gene expression[71]. Research has found that the spatial distribution of reactive gliosis in astrocytes coincides closely with the location of Aβ plaques in the brains of AD patients [72]. As typical markers for the immunohistochemical identification of astrocytes, glial fibrillary acidic protein (GFAP) and vimentin are expressed during reactive gliosis [73]. They play a role in the local regulation of Aβ pathology, capable of detecting or predicting Aβ deposition, which indicates cognitive decline [74]. Reactive astrocytes mediate the generation of Aβ. Astrocytes express a range of inflammatory factors, causing elevated levels of BACE and APP expression. APP is cleaved by α, β, and γ secretases, ultimately leading to the amyloidogenic degradation and generation of Aβ. When GFAP is overexpressed, the gliosis of reactive astrocytes worsens, potentially leading to glial scarring, which can disrupt neuronal structures and obstruct the ECS [75]. Reactive astrocytes also play a role in mediating the degradation of Aβ. On one hand, reactive astrocytes extend pseudopods to detach and phagocytize Aβ plaques from neurons; on the other hand, reactive glial cells can eliminate Aβ through degrading enzymes such as neprilysin or IDE [76, 77].

#### 4.1.2 Impaired Glymphatic Clearance of Aβ/Tau

Recent studies show that there exists a clearance system in the brain known as the Glymphatic System. It is prefixed with 'Glymphatic' because this system does not possess the lymphatic vessel structure composed of endothelial cells that the lymphatic system, in the narrow sense, has [78]. The Glymphatic System mediates the intracranial CSF-ISF exchange through a unique perivascular channel system formed by astrocytes, clearing abnormally accumulated proteins including Aβ and tau-proteins. It can also regulate the spatial distribution of non-toxic substances such as glucose, amino acids, and lipids in the brain, transporting ISF from the interstitial space to the veins, maintaining the patency of the interstitial space in the brain [78, 79]. Since the endfeet of astrocytes enclose 60-90% of the brain vasculature used for solute transport, astrocytes can dynamically regulate the interaction between neurons and brain vasculature, maintaining the stability of the glymphatic system to promote the clearance of metabolic waste such as Aβ [80]. However, astrocyte response to tissue damaging injury leads to anisomorphic astrogliosis, inhibits the transport function of the glymphatic system, weakens the clearance ability of metabolic waste such as Aβ in the ISF [79, 81, 82]. When the accumulated Aβ neurotoxic products exceed the astrocytes' loading capacity, it will lead to autophagy, which accelerates the accumulation of toxic proteins [83]. In addition to the glymphatic pathway, Aβ can also be eliminated through other complementary mechanisms, including enzymatic degradation by neprilysin and insulin-degrading enzyme [84], receptor-mediated transport across the blood-brain barrier like LRP1-mediated efflux [85], and microglial phagocytosis within the extracellular space [86]. These pathways, together with glymphatic flow, constitute an interconnected clearance network in which astrocytes, microglia, endothelial cells, and the extracellular space coordinate to maintain ISF homeostasis and limit toxic protein accumulation.

#### 4.1.3 AQP4 Mislocalization and Cytotoxic Edema

Since almost all synapses are enveloped by astrocytes, these cells can maintain homeostasis by regulating the production and clearance of interstitial fluid, ions, and neurotransmitters between synapses [6]. Among these, the water channel protein 4 in astrocytes (aquaporin-4, AQP4) plays a crucial role in the drainage of interstitial fluid [80]. In the brain, AQP4 is mainly distributed around blood vessels, subpial, and subependymal astrocytes, aggregating at astrocyte endfeet to form the glial limitans (serving as the central nervous system-cerebrospinal fluid interface). It participates in cerebrospinal fluid circulation, transports cerebrospinal fluid into the brain parenchyma, regulates cerebral blood flow and blood-brain barrier permeability, maintains the balance of water and ions in the brain, and plays a role in cytotoxic edema [87]. During the progression of AD, there is often an overexpression of AQP4, disruption of AQP4 localization in the cortex, increased permeability of the blood-brain barrier, and increased water permeability of the astrocyte cell membrane, leading to significant cytotoxic edema and even apoptosis. Studies have found that the abnormal distribution of AQP4 in astrocyte endfeet around cerebral cortical blood vessels is closely related to the formation of amyloid-beta (Aβ) and neurofibrillary tangles [88, 89] (Fig. 3).

**Figure 3.**
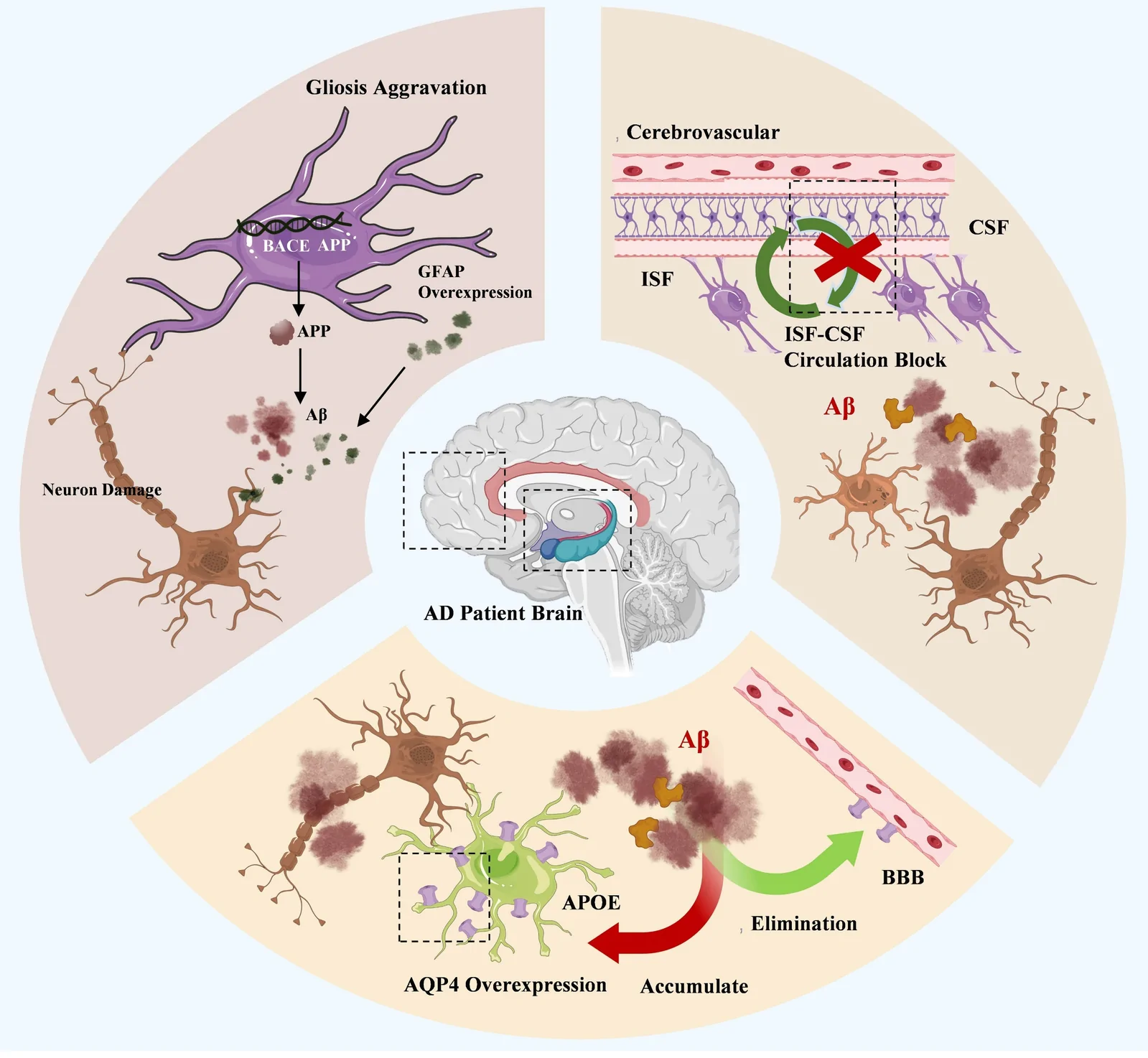
**Alterations of astrocytes in the brains of AD patients**. Upregulation of BACE and APP promotes Aβ production. GFAP overexpression induces reactive astrogliosis and glial scar formation. Astrocyte dysfunction disrupts CSF-ISF exchange, impairing glymphatic clearance of Aβ. AQP4 is overexpressed in glial cells, but its mislocalization in the cortex and increased BBB permeability lead to enhanced astrocytic membrane water permeability, resulting in cytotoxic edema and even apoptosis, thereby exacerbating neuronal loss. Abbreviations: AD: Alzheimer’s Disease; GFAP: Glial Fibrillary Acidic Protein; CSF: Cerebrospinal Fluid; ISF: Interstitial Fluid; BBB: Blood-Brain Barrier; AQP4: Aquaporin-4.

### 4.2 Microglia in AD

Microglia constantly interact with synapses throughout the lifespan, contributing to synaptic homeostasis and function. Several immune and neuronal proteins have been identified as potential upstream regulators of microglia-mediated phagocytosis and C1q production in AD models [90-92]. By pruning synapses, microglia may help protect neural circuits, alleviate excitotoxicity in the early stages of AD, and slow the progression of cognitive impairment [93] (Fig. 4).

**Figure 4.**
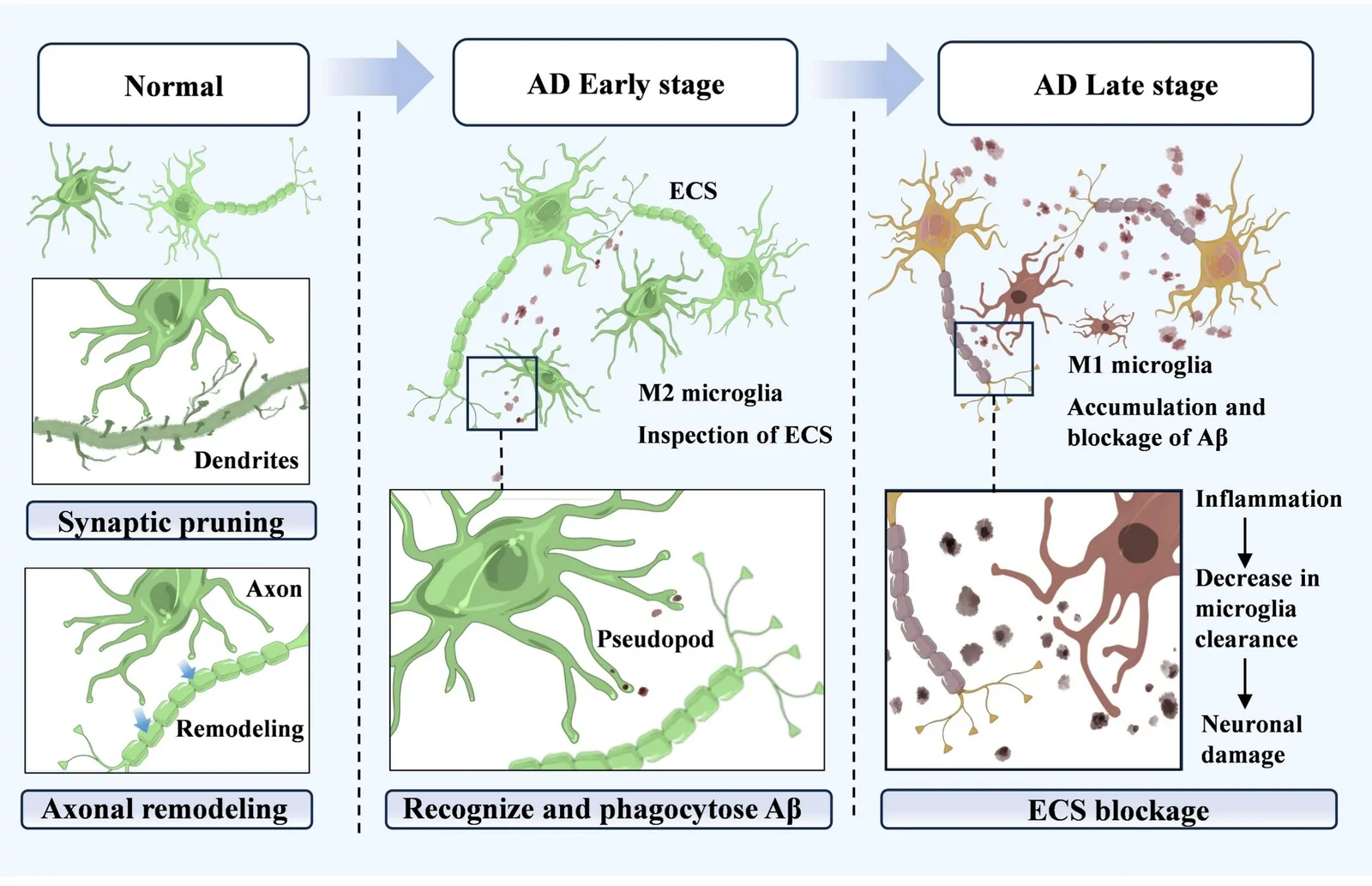
**Pathological changes of microglia in AD**. Under physiological conditions, microglia are involved in synaptic pruning, axonal remodeling, etc. In the early stage of AD, the M2 subtype differentiated from microglia is involved in recognizing and phagocytose Aβ. In the late stage of AD, inflammatory response and massive aggregation of Aβ lead to microglial apoptosis and blockage of extracellular interstitial fluid. Abbreviation: AD: Alzheimer’s Disease; ECS: extracellular interstitial fluid.

During the progression of Alzheimer’s disease (AD), microglia exhibit dynamic and heterogeneous activation patterns. Historically, these patterns were simplistically classified into M1 (neurotoxic) and M2 (neuroprotective) states. However, microglia with neuroprotective phenotypes gradually shift toward neurotoxic phenotypes as AD advances [93, 94]. Given that this dichotomous M1/M2 paradigm oversimplifies the complex activation states observed in vivo, it is now considered obsolete.

Human microglia respond to a wide array of environmental cues within the AD brain. Under neurodegenerative conditions, they undergo phenotypic changes characterized by enhanced phagocytic activity and pro-inflammatory responses. The disease-associated microglia (DAM) state is now recognized as a major microglial phenotype induced by the accumulation of damage-associated signals, including dying cells and misfolded proteins [95]. In the early stages of AD, microglia primarily adopt the DAM phenotype. As the disease progresses, multiple factors—including chronic type I interferon (IFN-I) signaling [96], metabolic dysfunction [97], persistent TREM2 signaling [98], and cellular senescence [99], adversely affect microglial phenotype, thereby limiting their reparative capacity and promoting dysfunction and senescence.

A recent single-nucleus RNA-sequencing (snRNA-seq) study using fresh surgical biopsies from patients with Aβ plaques and phosphorylated tau pathology mapped the transcriptional landscape of AD across different disease stages. This study identified three AD-associated microglial states related to autophagy, sphingolipid signaling and extracellular matrix organization, and antigen processing and lysosomal function [100].

Beyond the classical DAM state, microglia in neurodegenerative diseases display additional phenotypes, including interferon-response microglia (IRM), cycling/proliferating microglia, and antigen-presenting microglia (MHC II/LADAM) [101, 102]. IRM, for example, play a role in normal cortical development by phagocytosing neurons [103]. However, in pathological conditions such as AD, Parkinson’s disease (PD), and amyotrophic lateral sclerosis/ frontotemporal dementia (ALS/FTD), their overactivation can drive excessive synaptic pruning and neurodegeneration[104-106].

Moreover, microglia closely interact with the peripheral immune system. For instance, in a tauopathy model, T cells within the brain parenchyma exhibited clonal expansion and progressive exhaustion [107], suggesting that adaptive immune responses play a pivotal role in modulating microglial activity and disease progression.

In summary, microglial development follows a complex and tightly regulated sequence driven by environmental cues and linked to specific gene expression programs [108, 109]. These findings underscore the need for more refined characterization of microglial states in the human AD brain, which will be critical for understanding their diverse roles in disease pathogenesis and therapeutic targeting.

### 4.3 Oligodendrocytes in AD

The human brain myelin model indicates that the dynamic regulation process of myelin sheath generation, maintenance, repair, degradation, and decomposition is associated with the occurrence and development of degenerative diseases such as AD. It is also noted that during the process of homeostatic myelin repair, by-products such as Aβ and tau protein deposits are produced [110]. The 3xTg-AD mouse model is a widely used transgenic model for Alzheimer's disease. Comparing NG2 and myelin basic protein (MBP) in the hippocampus of 3xTg-AD mice and non-transgenic controls shows that at 6 months, oligodendrocyte precursor cells (OPCs) in 3xTg-AD mice have significant morphological atrophy and reduced MBP. By 24 months, these OPCs display hypertrophy, with increased cell surface area, volume, and enlarged soma and main process branches [111]. In addition to morphological changes, the increased deposition of Aβ plaques in the 3xTg-AD group is also noteworthy [112]. Therefore, in the early stages of AD, the disruption of OPCs and the deposition of Aβ plaques around them are typical pathological changes, which can lead to myelin damage and degradation, impairing cognitive abilities. In the mid-stage of AD, oligodendrocytes may release more cytokines, exacerbating neuroinflammation. As the disease progresses, ongoing neuroinflammation and oxidative stress may lead to the death of oligodendrocytes and also contribute to the disruption of the blood-brain barrier [113].

## 5. Mechanisms of Glial-Neuron Communication Disorders

### 5.1 Toxic Effects of Reactive Astrocytes on Neurons

Under pathological conditions, astrocytes become activated and transform into reactive astrocytes. Reactive astrocytes can respond to chemokines produced by neurons, expressing inflammatory cytokines, chemokines, ATP, excitatory amino acids, and other neurotoxic substances.

Researchers conducted mass spectrometry analysis on the proteins in the conditioned medium of reactive astrocytes and found an increased abundance of the lipoproteins APOE and APOJ in reactive astrocytes [114]. When reactive astrocytes secrete APOE, ATP binds with cassette transporters (ABCA1 and ABCG1) on the cell surface. Subsequently, they transport cholesterol and phospholipids, which combine with APOE to form phospholipid-protein particles. These particles then bind to A POE receptors on the cell surface, facilitating the redistribution of cholesterol and phospholipids on neurons, thereby completing lipid metabolism [115]. APOE not only mediates lipid metabolism but also regulates synaptic plasticity and promotes or clears Aβ [116]. Knocking out the metabolic enzyme ELOVL1, which is responsible for the synthesis of long-chain, fully saturated lipids (≥ C16:0) in reactive astrocytes, significantly reduces the toxicity of these astrocytes to neurons in vitro. This indicates that reactive astrocytes can secrete toxic lipids, such as APOE and APOJ, to drive neuron death [114] Through lipidomics analysis to quantify cholesterol esters in postmortem human brains and induced pluripotent stem cell (iPS)-derived cells, researchers found that cells with APOE4 had relatively lower cholesterol efflux, with most cholesterol being stored within the cells. This condition leads to difficulties in myelin sheath formation, further causing abnormal neuron function [117].

Previous study has found that APOE4 is a strong driver of Aβ accumulation, and this Aβ accumulation leads to neuron function impairment, triggering a toxic cascade reaction [117-119]. Pathological studies of the brain tissue of deceased AD patients show that APOE4 promotes Aβ aggregation, exacerbating the deposition of plaques in the brain parenchyma. Using in vivo microdialysis, researchers analyzed the clearance kinetics of Aβ after halting its production and concluded that APOE4 can impair the clearance of Aβ by ISF [116, 120]. ISF clears soluble Aβ in an APOE isoform-dependent manner, so the isoform and lipidation state of APOE can affect the clearance of Aβ from ISF, thereby causing communication disorders in the brain [115].

By measuring signals related to astrocyte-neuron energy metabolism and transmission through functional magnetic resonance imaging (fMRI) [121], it has found that in normal conditions, neuronal excitation generates glutamate, prompting astrocytes to increase glucose uptake and glycogen breakdown. This process supplies neurons with energy and metabolites like L-lactate and L-serine, supporting their energy needs [122, 123]. Pathological damage can disrupt the metabolic coupling between astrocytes and neurons. Impaired astrocyte reuptake can lead to glutamate's neurotoxic effects. Aβ disrupts neuronal Ca^2+^ balance and mitochondrial function, affecting glutamatergic transmission via NMDAR and mGluR. It also boosts glutamate release from neurons and astrocytes while lowering EAAT expression and function, raising extracellular glutamate levels. This activates postsynaptic NMDAR, triggering excitotoxicity (Fig. 5).

**Figure 5.**
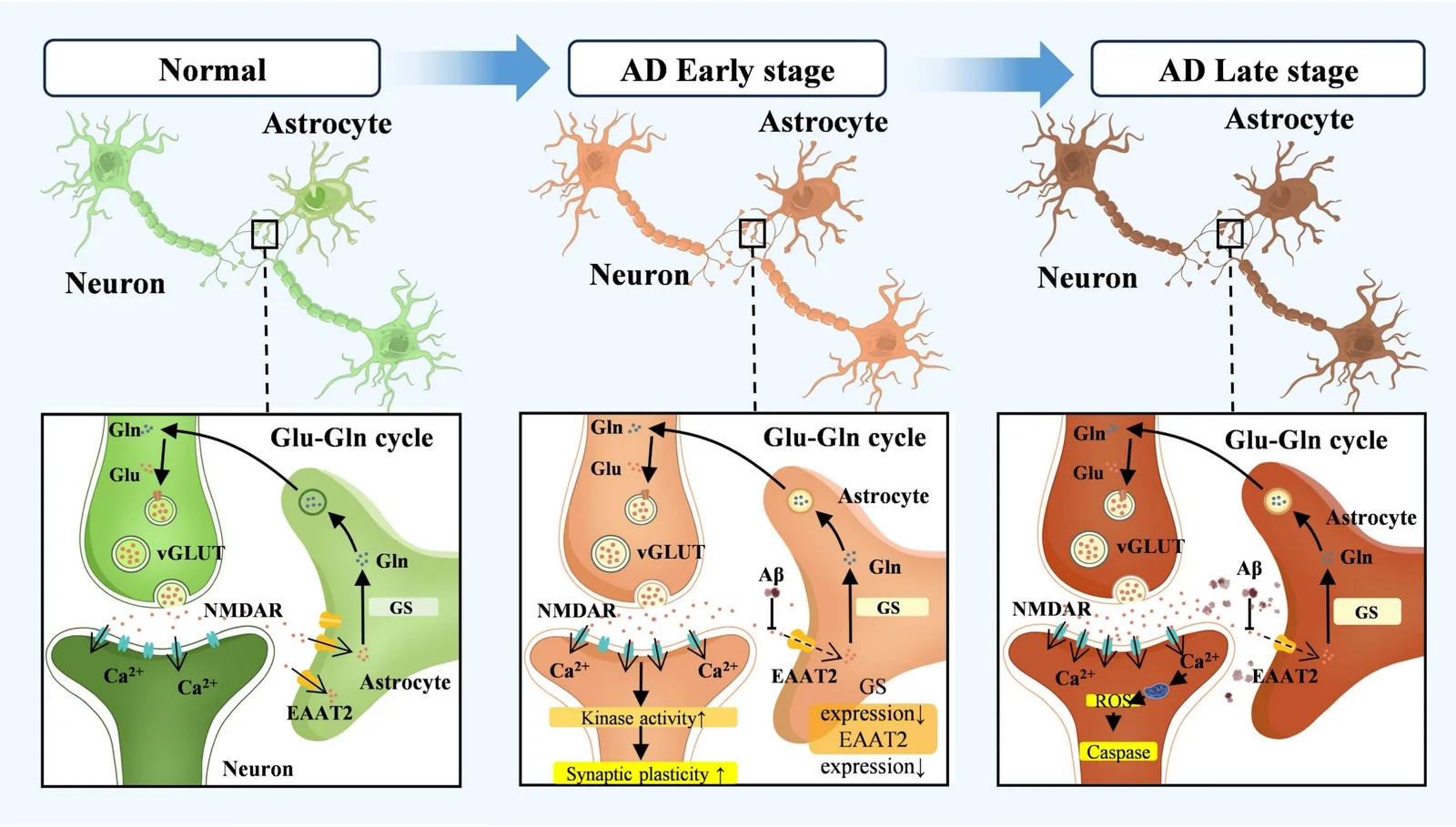
**Pathological changes of astrocytes in AD**. Under physiological conditions, astrocytes are involved in the glutamate cycle between neurons. In early AD, Aβ formation impairs EAAT2 expression, while NMDAR activity increases compensatory, enhancing synaptic plasticity. In late-stage AD, Aβ fully inhibits EAAT2 on astrocytes, disrupting the glutamate cycle. Excessive reactive oxygen species are released from postsynaptic neurons, leading to neuronal necrosis. Abbreviations: AD: Alzheimer’s Disease; Glu: Glutamate; vGLUT: Vesicular Glutamate Transporter; GS: Glutamine Synthetase; ROS: Reactive Oxygen Species.

In AD patients, Aβ accumulation in astrocytes impairs intercellular communication, altering energy metabolism and antioxidant functions. Elevated β-APP levels lead astrocytes to internalize Aβ, increase copper reduction, and produce more reactive oxygen species, worsening mitochondrial DNA damage. This imbalance heightens oxidative stress in astrocytes. This is accompanied by Aβ-induced dysfunction in glucose metabolism, disruption of the pentose phosphate pathway, and impairment of the reactive oxygen species detoxification pathways involving NADPH and GSH, leading to a metabolic coupling imbalance between astrocytes and neurons [124-127] (Fig. 6A).

### 5.2 Tau-Mediated Microglia Overactivation

Tau protein is primarily expressed in neurons, where under physiological conditions, it binds to microtubules to stabilize the cytoskeleton [128]. When tau protein is excessively phosphorylated, it undergoes abnormal folding, depositing within neurons to form oligomers [129]. When the Tau protein accumulates to a certain level within the neuron, the cell produces a large amount of reactive oxygen species, which activate the phosphatidylserine (PS) transporter, conveying the ‘eat me’ pro-phagocytic signal PS [130, 131]. When microglia contacts to this pro-phagocytic signal, they become activated, triggering phagocytosis, and rapidly engulf the neuron by releasing opsonins. Opsonin (such as MFGE8), as a soluble secreted protein, can simultaneously bind to the pro-phagocytic signal phosphatidylserine on neurons and the αvβ3 phagocytic receptor on microglia, causing cytoskeletal rearrangement and enhancing phagocytosis [132, 133]. Due to the overactivation of microglia, communication between microglia and neurons becomes disordered. Opsonin induces the production of the pro-inflammatory mediator NO. Meanwhile, the iNOS/NO signaling pathway promotes the release of MFGE8, which feeds back to the αvβ3 receptor of microglia to further amplify the iNOS/NO signaling pathway, exacerbating the inflammatory response, creating a cytotoxic environment, and intensifying the vicious cycle of Tau pathology [133] (Fig. 6B).

**Figure 6.**
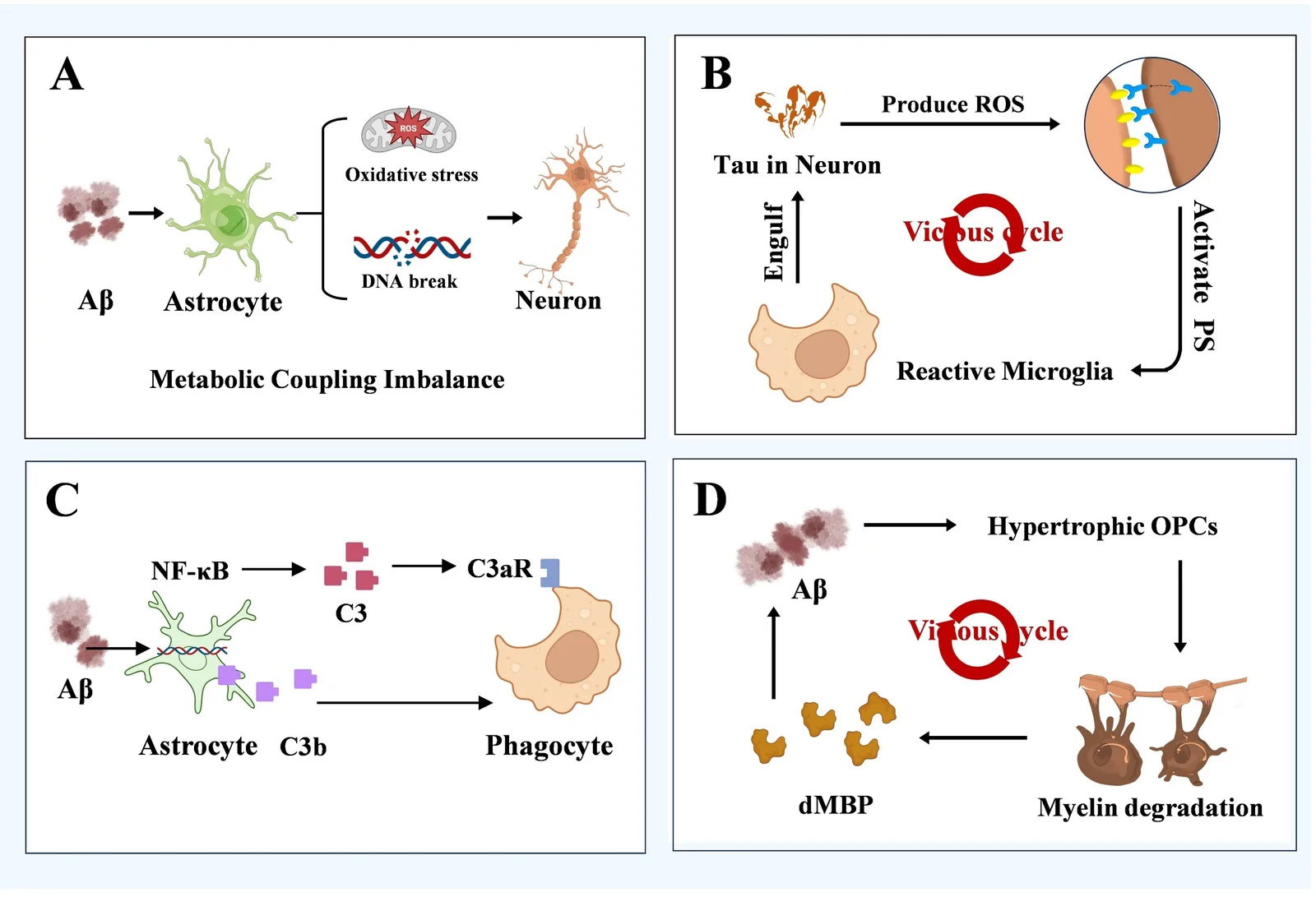
**Mechanisms of glial communication dysfunction**. (**A**) Astrocytes produce excessive reactive oxygen species, leading to DNA damage and impairment of antioxidant systems such as NADPH, resulting in disrupted metabolic coupling with neurons. (**B**) Intraneuronal accumulation of Tau protein upregulates phosphatidylserine expression, triggering microglial phagocytic activity. Additionally, microglia release pro-inflammatory mediators such as nitric oxide, further perpetuating Tau pathology. (**C**) Aβ activates the NF-κB pathway in astrocytes, inducing C3/C3b release and interaction with C3aR, which promotes microglial phagocytosis of Aβ and exacerbates AD pathology. (**D**) Degradation products of oligodendrocytes, particularly damaged myelin basic protein, contribute to myelin degeneration and deposit within amyloid plaques, enhancing Aβ toxicity. Abbreviations: ROS: Reactive Oxygen Species; PS: Phosphatidylserine; NO: Nitric Oxide; iNOS: Inducible Nitric Oxide Synthase; dMBP: Damaged Myelin Basic Protein.

### 5.3 Astrocyte C3-Microglial C3aR Axis in Aβ Pathology

The communication disorder between astrocytes and microglia is primarily mediated by the astrocyte C3/c3a - microglia C3aR axis. Aβ stimulates NF-κB in astrocytes, inducing them to secrete C3. Once C3 binds to C3aR, it can induce microglia to phagocytize Aβ protein, exacerbating Aβ pathology [134]. Additionally, the transmission of C3b through astrocyte-derived exosomes can also provoke microglia-induced neurotoxicity [135]. Microglia express various receptors that directly respond to amyloid protein deposition. For example, after CD36 recognizes β-amyloid peptides, its Src kinase signals trigger the assembly of a novel heterotrimeric complex CD36-TLR4-TLR6, facilitating the recruitment of microglia to plaque deposits. Meanwhile, this signaling propagates through MyD88 and TRIF adapters, inducing neurotoxicity [136] (Fig. 6C).

### 5.4 OPC-Linked Aβ Deposition

As the 3xTg-AD mice age, the pathological changes in their AD-related brain regions progressively worsen [112]. In 24-month-old 3xTg-AD mice, immune-fluorescence labeling reveals hypertrophic OPCs densely arranged between Aβ plaques, which gradually surround and even embed into the plaques when observed under a microscope. The volume and surface area of hypertrophic OPCs increase, myelin is damaged, and the degraded damaged myelin basic protein (dMBP) increases, colocalizing with the aβ1-42 core of the amyloid plaques [137]. These pieces of evidence suggest that axonal damage leading to myelin damage results in the deposition of dMBP in amyloid plaques, exacerbating the toxic effects of amyloid proteins. The stagnation of axonal transport also intensifies the interaction between BACE1 and APP within axonal transport vesicles, promoting the deposition of amyloid proteins [138]. On the other hand, damage to myelin and axonal injury impedes the communication functions between neurons, reducing the capacity for intercellular information transmission and leading to a decline in learning and memory abilities (Fig. 6D).

## 6. Downstream Pathways to Neuronal Death

### 6.1 Immune Signaling and Selective Vulnerability

The Gladstone Institutes utilized the Kyoto Encyclopedia of Genes and Genomes (KEGG) [139], to study which pathways are specifically related to the expression levels of APOE in neurons. The results show that the expression levels of neuronal APOE are linked to immune response pathways [140]. In Alzheimer's patients, neurons overexpress APOE, which increases MHC-I gene expression. MHC-I regulates and can eliminate neuronal synapses via immune pathways, and its overexpression suggests an excessive immune response, leading to selective neuron degradation [140].

### 6.2 Oxidative stress leads to neuron death

The brain's high oxygen demand and limited antioxidants make it vulnerable to damage from reactive oxygen species. In Alzheimer's disease, Aβ accumulation triggers toxic stimuli, excessive NO production, PARP-1 activation, oxidative stress, and rapid depletion of NAD+ and ATP. This harms neurons, disrupts hippocampal synapses, and impairs cognition [141, 142]. When a large amount of reactive oxygen species is produced within the cell, it triggers an oxidative stress response, leading to DNA peroxidation and damage. This excessively activates PARP-1, consuming large amounts of NAD+, hindering mitochondrial ATP synthesis, and rapidly depleting energy within neuronal cells, ultimately resulting in PARP-1 dependent cell death [143-145]. Abnormal Aβ accumulation disrupts astrocyte-neuron metabolic coupling and reactive oxygen species detoxification, causing oxidative damage, cytotoxicity, and increased neuron susceptibility to ferroptosis [146]. As a lipid-based form of reactive oxygen species, the accumulation of phospholipid hydroperoxides can very quickly damage the neuronal cell membrane, which cannot be repaired, leading to cell death [147].

**Figure. 7:**
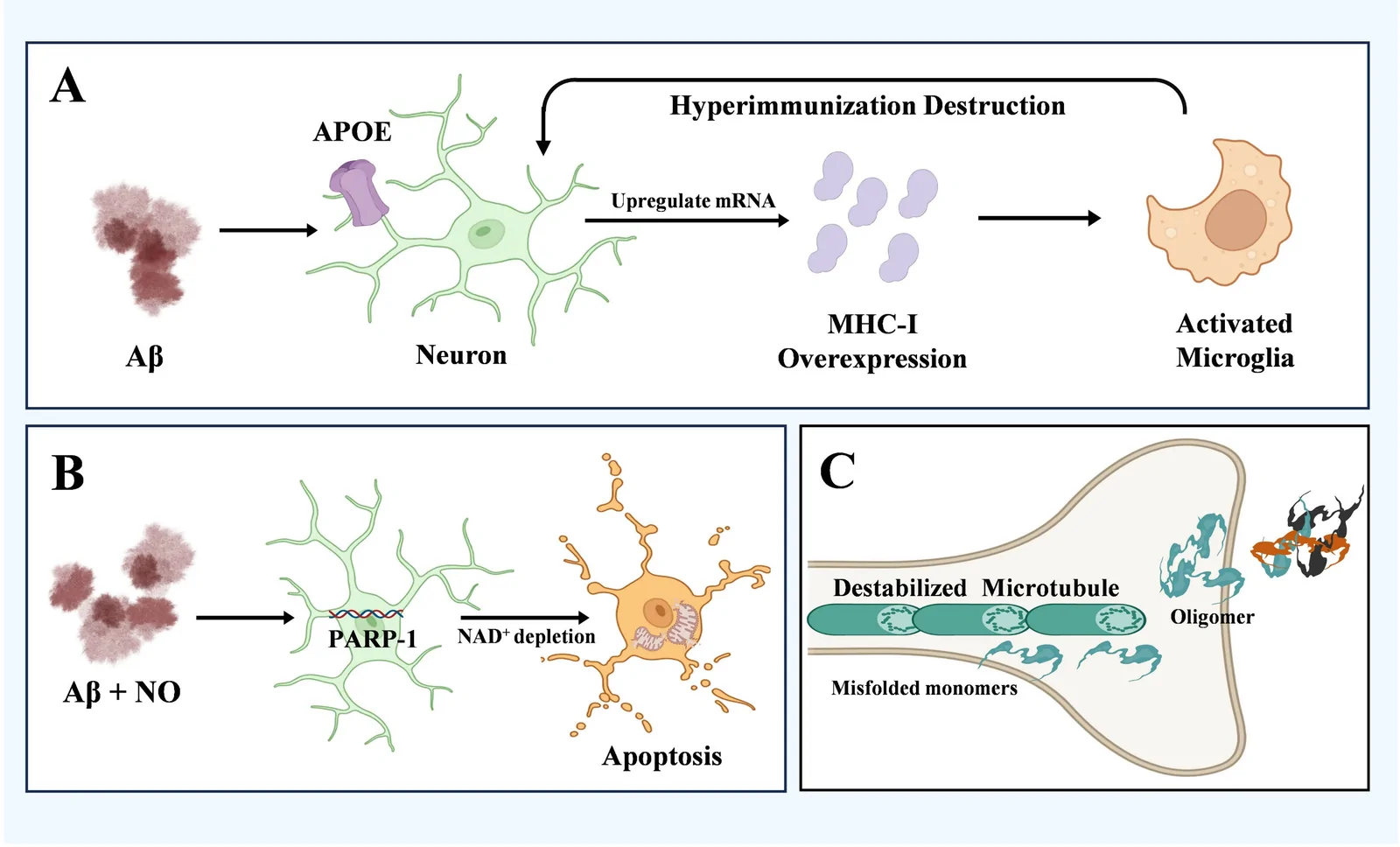
**Pathways of neuronal apoptosis induced by disrupted communication between neurons and glial cells**. A. Upregulated expression of aquaporins at the mRNA level enhances MHC-I expression, triggering excessive immune responses. B. Aβ accumulation and excessive nitric oxide production activate PARP-1, leading to cell death. C. Tau proteins, detached from microtubules, form oligomers that progressively accumulate intracellularly into insoluble neurofibrillary tangles, ultimately resulting in neuronal death. Abbreviations: AD: Alzheimer’s Disease; Glu: Glutamate; APOE: Aquaporin; NO: Nitric Oxide.

### 6.3 Tau-Dependent Neurotoxicity

In the progression of AD, abnormalities occur in the post-translational modification of Tau protein, often resulting in hyperphosphorylation [148]. At this point, the previously positively charged microtubule-binding region loses its positive charge and detaches from the negatively charged tubulin, resulting in the disassembly of microtubules [149]. Destruction of the microtubule structure disrupts its stabilizing and transport roles, leading to neuronal death. Detached Tau protein aggregates into oligomers and accumulates to form insoluble neurofibrillary tangles [150]. As the disease progresses, these neurofibrillary tangles increase, gradually enveloping neurons, impairing normal cellular physiological functions, and ultimately leading to neuronal death [151].

Previous studies suggested that microglia, as immune cells, can phagocytize Aβ to maintain homeostasis and also neurons under pathological conditions, causing neuronal death [152]. Neurons exposed to phosphatidylserine send pro-phagocytic signals, while M1-type microglia release opsonin for phagocytosis. Additionally, Tau protein can transfer from diseased to healthy neurons via synapses [153], thus, Tau protein-induced neuronal death develops into a vicious cycle in the brains of AD patients, continuously exacerbating irreversible neural damage (Fig. 7).

## 7. New Insight into AD onset and Treatment Strategies

### 7.1 Formaldehyde (FA) as a Novel Target

Alzheimer’s disease (AD) is characterized by multiple well-established pathological hallmarks, most notably the extracellular accumulation of Aβ forming amyloid plaques, and the intracellular aggregation of hyperphosphorylated Tau protein leading to neurofibrillary tangles (NFTs). In addition to these core mechanisms, other processes such as neuroinflammation, oxidative stress, and impaired clearance pathways have also been implicated in AD pathogenesis.

Beyond these well-established pathways, recent studies have proposed formaldehyde (FA) as a potential molecular contributor to Alzheimer’s disease (AD) pathology. FA is a colorless, volatile, and toxic gas that also occurs endogenously within the cytoplasm, nucleus, and subcellular organelles of living cells, where it may function as a gaseous signaling molecule involved in memory regulation [154, 155].

Experimental evidence from transgenic AD mouse models has shown significantly elevated FA concentrations in the brain [156], while clinical studies have reported increased FA levels in the urine of AD patients, which appeared to correlate with dementia severity [156, 157]. These findings suggest a possible association between endogenous FA levels and cognitive decline, though the relationship remains correlational rather than causal.

Mechanistically, in vitro and in vivo studies indicate that excessive FA may promote Aβ aggregation by facilitating the cross-linking of Aβ monomers, particularly at the K28 residue, thereby accelerating the formation of Aβ oligomers and fibrils [158, 159]. In late-stage AD patients, the excessive accumulation of endogenous FA can promote the formation of Aβ and Tau protein aggregation [160, 161], which block the brain ECS and obstruct the drainage of brain ISF [162]. Moreover, FA has been reported to activate astrocytes and microglia in wild-type mice, increasing oxidative stress and the production of pro-inflammatory cytokines, which may disrupt glia-neuron communication and further contribute to neurodegenerative processes [163].

Despite these intriguing findings, critical gaps remain. Most evidence linking FA to AD pathology is derived from animal models and cell-based studies, with only limited observational data available from humans. The spatiotemporal distribution of FA in the human brain remains poorly characterized, and its apparent enrichment in regions such as the hippocampus and cerebellum [164] does not by itself demonstrate a causal role in AD pathogenesis.

### 7.2 Potential of Astrocytes as New Targets for Therapeutic Intervention

During the progression of AD, astrocytes undergo morphological and functional disruptions, leading to neuronal damage and even death. Consequently, a growing number of studies are focusing on astrocytes to identify new targets for AD treatment [5].

One promising therapeutic avenue involves modulating astrocyte activity to restore healthy communication within the neural network and improve the brain’s microenvironment. Both genetic and pharmacological approaches have been explored to achieve this. For instance, increasing IL-33 expression in astrocytes boosts synapse pruning signals to microglia, aiding in the efficient removal of excess synapses [27]. In a complementary line of work, Benjamin L. L. Clayton and others developed a high-throughput screening platform to model AD-related astrocyte pathology and identify compounds capable of reducing astrocyte reactivity [165, 166]. Among these, histone deacetylase 3 (HDAC3) inhibitors emerged as promising candidates. By inhibiting HDAC3, these compounds can downregulate reactive astrocyte genes through modulation of RelA/p65 signaling [167], while simultaneously inducing neuroprotective NRF2 target genes [168], thereby reducing gliosis and supporting neuronal survival [169, 170]. Conversely, enhancing histone acetylation through histone acetyltransferase activity may promote memory formation by facilitating transcription, with cAMP response element-binding protein (CREB) playing a pivotal role in long-term memory processes [171]. These findings suggest that fine-tuning epigenetic regulation in astrocytes could offer a dual benefit: mitigating harmful reactivity while enhancing cognitive function.

Another strategy focuses on leveraging astrocytes’ intrinsic ability to clear Aβ, a central driver of AD pathology [172]. In experimental models, astrocytes have been shown to migrate toward Aβ deposits, engulf them, and degrade Aβ in the presence of APOE [173]. Genetic modification of astrocytic APOE has been proposed as a way to enhance this clearance process, potentially slowing disease progression and improving the neuronal survival environment.

However, most of these findings are derived from animal or in vitro models, and the translational relevance to human AD pathology remains uncertain; moreover, astrocyte heterogeneity and context-dependent functions may limit the generalizability of these therapeutic approaches [174, 175].

### 7.3 Microglia-Modulating Approaches

Over the past ten years, a lot of studies on microglia for Alzheimer's treatment have mainly targeted reducing neuroinflammation. Early clinical trials using anti-inflammatory agents, such as cyclooxygenase inhibitors (e.g., celecoxib and naproxen) or aspirin, aimed to reduce inflammatory damage and improve cognition, but these approaches have not produced significant clinical benefits [176-179]. This has shifted the focus from broadly dampening inflammation to modulating microglial function in a more nuanced manner. Given microglia’s dual role—both contributing to neurotoxicity and clearing protein aggregates—emerging strategies now aim to reprogram microglia from a detrimental to a neuroprotective phenotype [180].

Preclinical studies suggest that promoting this phenotype switch can enhance the clearance of amyloid plaques and cellular debris, thereby preserving neural circuits [181]. For example, CHF 5074 has been shown to activate TREM2-dependent pathways and drive microglia toward a reparative state, facilitating Aβ clearance and circuit remodeling [182, 183]. Parallel efforts are investigating key signaling and cytokine pathways that regulate microglial polarization, with the goal of identifying druggable targets [180, 184-186].

However, Most evidence for phenotype switching and therapeutic benefit comes from cell or animal models; the translational validity of these mechanisms in human Alzheimer’s pathology has yet to be established [187, 188]. Thus, further research is needed to precisely define disease-relevant microglial subpopulations and to validate their modulation in clinical settings.

### 7.4 Oligodendrocyte-Directed Strategies

Myelin dysfunction models show that aging-induced structural defects and damage in myelin directly or indirectly promote the formation of amyloid plaques. Consequently, myelin damage is identified as an upstream risk factor for AD [189]. Therefore, targeting oligodendrocytes to enhance their health and myelin integrity may represent a novel approach to slowing the progression of AD. In 2019, Peisu Zhang proposed an anti-aging therapy, which involves a cocktail treatment using Dasatinib and Quercetin (D+Q). The results demonstrated that this method can eliminate senescent OPCs, reduce amyloid plaque deposition in AD mice, and improve cognitive deficits [190]. While animal studies suggest oligodendrocyte involvement in AD, the extent and nature of such pathology in humans remain debated.

### 7.5 ECS-Restoring Therapies

#### 7.5.1 Phototherapy to Reopen ECS and Restore Communication

Current research indicates that the abnormal deposition of Aβ in the hippocampus and cortex of the brain is a core pathological feature of AD [191, 192]. Aβ deposition triggers a series of cascade reactions, including the abnormal hyperphosphorylation of Tau protein, neurotransmitter disruption, and oxidative stress [193-195]. Therefore, preventing Aβ deposition is considered one of the most reliable treatment strategies [196]. However, despite extensive drug development efforts, most clinical trials targeting Aβ have failed to produce meaningful clinical benefits [197]. One potential explanation is that Aβ plaques accumulate within the extracellular space (ECS), physically obstructing interstitial fluid (ISF) flow and limiting the delivery of drugs to deeper brain regions [198]. These limitations have prompted exploration of alternative, non-invasive therapeutic strategies, among which phototherapy has emerged as a promising approach due to its multifaceted mechanisms and accessibility.

Phototherapy may improve both the biological environment of the brain and AD-related symptoms through complementary pathways. Clinically, light exposure has been shown to stabilize circadian rhythms, improving sleep quality and reducing agitation and aggression in patients [199-201]. At a cellular level, phototherapy appears to mitigate oxidative stress and inflammation, thereby protecting neurons from damage and supporting overall brain health [202].

In the treatment of AD animal models, phototherapy has been found to directly or indirectly clear Aβ from the brain. Particularly, the therapeutic effects of red and near-infrared light are more significant [203]. For instance, 670 nm red light treatment in APP/PS1 mice reduces Aβ burden in the hippocampus and cortex while alleviating oxidative stress-induced neuronal damage [204]. Similarly, 1070 nm near-infrared light activates microglia to enhance Aβ clearance, leading to improved cognitive performance [205]. These effects are thought to arise from multiple, interconnected mechanisms: reducing Aβ aggregation, improving synaptic function, enhancing ISF drainage, and restoring glymphatic clearance [206-208]. Interestingly, specific wavelengths such as 630 nm red light can also modulate related molecular pathways by directly inhibiting Aβ aggregation and activating formaldehyde dehydrogenase (FDH) to degrade endogenous formaldehyde (FA), thereby reducing FA-induced Aβ aggregation [198]. In summary, phototherapy has made some progress in the treatment of AD, but more research is needed to further promote its application in clinical practice, with the hope of providing better treatment outcomes for patients (Table 1).

**Table 1:** Summarizes current agents for AD, their mechanisms of action, clinical trial details, phase of development, and common adverse events.

Agent	Mechanisms of action	Clinical trial	Phase	Common adverse reaction events	Ref.
**Donanemab**	Targets pyroglutamate modified Aβ Promotes plaque clearance	NCT05026866	Phase 3	Amyloid-related imaging abnormalities	[209, 210]
**GV1001**	Promotes microglia phagocytosis Clearance of Aβ plaques	NCT05189210	Phase 3	Amyloid-related imaging abnormalities Infusion-related reaction	[211] [212]
**E2814**	Inhibits tau aggregation	NCT01760005 NCT05269394	Phase 3 Phase 3	Amyloid-related imaging abnormalities Infusion-related reaction	[213]
**Fosgonimeton**	Inhibits the release of impact foctors Improves synaptic plasticity Reduces neuronal damage	NCT04488419 NCT04886063	Phase 3 Phase 3	Injection site pain Injection site pruritus	[214, 215]
**SHR-1707**	Block the formation of Aβ plaques Promotes the microglial phagocytosis	NCT06114745	Phase 1	Dysgeusia and fatigue	[216, 217]
**Remternetug**	Recognizes Aβ pyroglutamated form	NCT05463731	Phase 3	Amyloid-related imaging abnormalities Infusion-related reaction	[218, 219]
**Posdinemab**	Binds pathological phosphorylated tau Neutralize pathologica tau	NCT04619420	Phase 2	Post-LP syndrome	[220]
**Lecanemab**	Binds to large soluble Aβ fibril	NCT01767311 NCT03887455	Phase 2 Phase 3	Amyloid-related imaging abnormalities Infusion-related reaction	[221, 222]
**L-Serine**	Reduces inflammation by M2 microglia Activates the glycine receptor	NCT03062449	Phase 2	longer time to diffuse through the blood-brain barrier and might even engender nephrotoxicity	[223]
**PRI-002**	directly destroys toxic and replicates Aβ	NCT06182085	Phase 2	EEG abnormalities	[224]

#### 7.5.2 ECS-Guided Drug Delivery

The brain ECS is an ultrastructural space located between neurons and between neurons and blood vessels, accounting for about 20% of brain tissue volume. It contains interstitial fluid, extracellular matrix, and various nutrients, ions, and neurotransmitters required by neurons [172, 225]. In AD, the balance of biomolecules in the brain ECS is disrupted, leading to neuronal damage and dysfunction [226]. Because the ECS provides a direct pathway for the exchange of substances, targeting this space for drug delivery offers a way to bypass the blood-brain barrier, delivering treatments directly to sites of pathology [227]. Thus, understanding and monitoring ECS changes is not only important for clarifying AD pathology but also critical for optimizing therapeutic strategies.

In recent years, significant progress has been made in ECS detection technologies, which can be broadly divided into three main methods. While each approach has unique advantages and limitations, combining their strengths offers a more comprehensive understanding of ECS dynamics in both health and disease [228-231].

The ion-selective microelectrode method employs highly selective ion microelectrodes to monitor the concentration changes of specific ions in the brain ECS in real time, thereby revealing various physiological and pathological processes of neurons [8]. The tips of these microelectrodes are only a few micrometers in diameter, allowing them to be directly inserted into brain tissue with minimal damage, thus measuring the ion concentrations in local areas, such as important ions like K^+^, Na^+^, and Ca^2+^ [232]. By monitoring the concentration changes of these ions in real-time, we can understand the activity state of neurons and the efficiency of synaptic transmission, providing crucial experimental evidence for studying the biomolecular imbalance in the ECS during AD [233]. However, because ion fluxes are highly complex and measurements are very localized, this method is most effective when integrated with imaging or MRI techniques to build a broader, systems-level view of ECS function [234]. Meanwhile, microelectrodes will age after being used for some time, requiring regular replacement.

Second, integrated optical imaging methods acquire three-dimensional structural and functional information of the brain ECS in a non-invasive manner by utilizing optical microscopes, optical sensors, and image processing algorithms [235]. This method can characterize distances within the ECS and reflect the behavior of proteins or macromolecular drugs in the ECS and their interactions with the extracellular matrix [236]. However, its depth of imaging is limited to superficial cortical regions (approximately 200 μm) [237], making it less effective for deeper brain structures [238]. To address this, optical imaging can be combined with other modalities, such as MRI, to bridge superficial and whole-brain observations, thereby providing a more complete spatial map of ECS dynamics.

**Figure 8.**
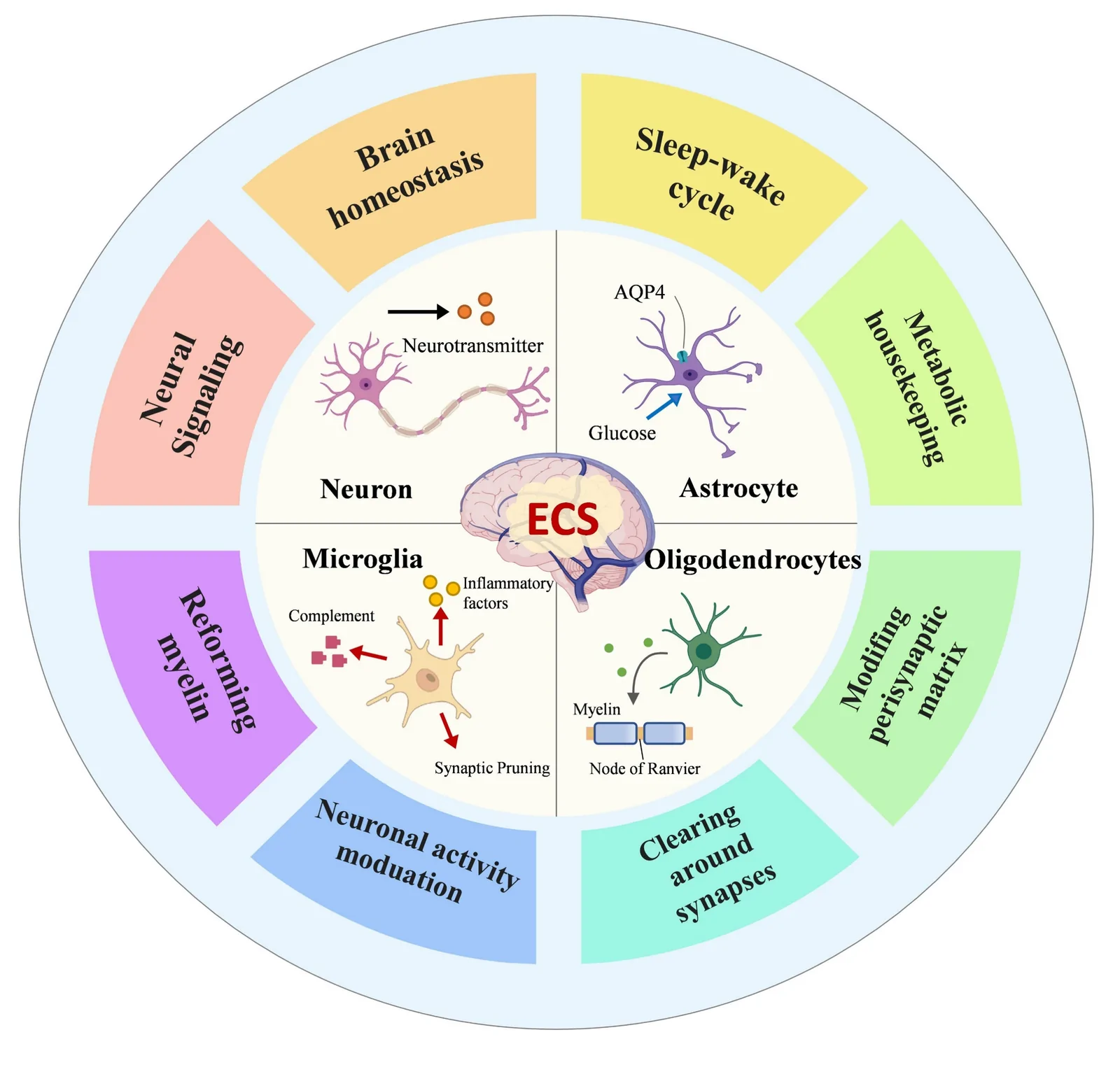
**The extracellular space (ECS) and its connections with neurons and glial cells**. The ECS is an essential component of the central nervous system. It participates in neuronal signal transmission and the maintenance of brain cell homeostasis. The ECS, through astrocytes, regulates the sleep-wake cycle and maintains cerebral metabolic balance. Through microglia, the ECS contributes to synaptic remodeling, neural plasticity, and the clearance of synaptic debris. Via oligodendrocytes, the ECS is involved in myelin remodeling and responses to neuronal activity. Abbreviations: ECS, extracellular space.

Magnetic resonance imaging (MRI), as a non-invasive technique, can measure the apparent diffusion coefficient of water molecules and tissue anisotropy in vivo, thereby reflecting the structural and dynamic information of the ECS [239]. Recent advancements, such as Gd-DTPA tracer-based MRI, have greatly enhanced detection depth and resolution, making it the only technique currently capable of visualizing the entire brain ECS in vivo [227].The principle of this method is that after the tracer injected into the body reaches the brain via blood circulation, it interacts with water molecules in the brain ECS, altering their local magnetic field environment [227]. When radiofrequency pulses are applied to these water molecules, they produce specific signal responses, which are captured by the MRI scanner and converted into images [239]. Furthermore, techniques like magnetic resonance spectroscopy (MRS) and functional MRI (fMRI) extend MRI’s capabilities, enabling the detection of metabolite concentrations and neuronal activity patterns, which together reveal both structural and functional aspects of the ECS [240, 241] (Fig. 8).

By integrating these three technologies—ion-selective microelectrodes for precise local measurements, optical imaging for real-time visualization, and MRI for whole-brain mapping—a multi-scale and multi-dimensional picture of ECS dynamics can be constructed. This comprehensive approach not only improves our understanding of ECS-related pathological changes in AD but also allows researchers to monitor how therapeutic interventions alter ECS structure and function. Such integration provides a rational basis for selecting and optimizing treatment strategies [242, 243] (Table 2).

**Table 2:** Summarizes therapeutic agents for Alzheimer’s disease targeting glial cells and the endocannabinoid system, outlining their mechanisms and outcomes.

Target	Agent	Mechanisms of action	Stage	Disease	Result	Ref.
Astrocyte	Arundic acid (AA)	a novel modulator of astrocyt	Phase 1	MCI	a favorable trend in reduction of NIHSS	[252]
Astrocyte	AST-004	a novel combined adenosine A1R/A3R receptor agonist.	Phase 1	MCI	safe and well-tolerated	[253]
Astrocyte	histone deacetylase 3 (HDAC3) inhibitors	inhibit the reactivity of astrocytes	Pre-clinical	AD	reducing gliosis, restore contextual memory	[174]
Microglia	AL002	an innate immune receptor expressed by microglia	Phase 1	AD	increases in biomarkers of TREM2 signaling, microglia recruitment	[254]
Microglia	CHF5074	a new microglial modulator	Phase 2	AD	No significant differences in neuropsychological tests	[255]
Microglia	MINocyclinE	reduce inflammation	Phase 2	AD	no effect on 11C-PK11195 binding (microglial signal)	[256]
Oligodendrocyte.	Dimethylsulfonio- propionate (DMSP)	a natural neuroprotective propertie	Pre-clinical	AD	myelin sheath restoration	[257]
Oligodendrocyte	Senolytic treatment	alleviates Aβ-associated oligodendrocyte progenitor cell senescenc	Pre-clinical	AD	alleviates cognitive deficits	[198]
ECS	630 nm red light (RL)	mashed Aβ deposition in the ECS, recovered the flow of ISF	Pre-clinical	AD	rescued cognitive functions in AD mice	[258]
ECS	electroacupuncture (EA)	enhance paravascular influx in the glymphatic system	Pre-clinical	AD	reduce Aβ accumulation	[259]
ECS	Transcranial magnetic stimulation (TMS)	Regulate glymphatic system	Pre-clinical	AD	reduced Aβ deposition and enhanced spatial memory cognition.	[260]

## 8. Outlook and challenges

This paper highlights the crucial roles of glial cells in the CNS, emphasizing their support and protection of neurons, stabilization of the microenvironment, immune response participation, and contribution to neural information networks under physiological conditions. At the same time, this paper reviews how communication issues between glial cells and neurons contribute to the onset and pathological progression of AD. In addition, we specifically discuss the extracellular space (ECS), the fundamental external environment for neurons and glial cells. It serves as the foundation for communication between neurons and glial cells, but under pathological conditions it is prone to disruption, thereby exacerbating the worsening of AD [254].

Initially, AD treatments primarily rely on cholinesterase inhibitors and excitatory amino acid receptor antagonists, aiming to enhance the action of cholinergic neurotransmitters in order to improve cognitive function [255]. But these approaches fall short in fully alleviating disease progression. At present, the most effective therapeutic strategy for AD is the use of anti-Aβ monoclonal antibodies [256], such as Donanemab, though their clinical application remains limited by high costs and the inability to reverse symptoms.

This review looks at current AD therapies that target glial cells. Most studies so far have focused on microglia, but none of these approaches have made it into real clinical use for AD [247]. Treatments aimed at astrocytes or oligodendrocytes are usually designed for stroke or multiple sclerosis (MS), and in AD they are still stuck at the animal study stage [245, 250]. We also discuss non-invasive physical therapies that act on the endocannabinoid system (ECS), though right now these can only be used as supportive treatments [251].

Due to the complex composition of glia and the complex modulation procedures involving multiple signal molecules, current studies are sometimes heterogeneous. In future, more methodologies should be developed, such as long-term or high-resolution imaging, to observe the interaction between glia and neurons, especially with regard to the progression of Aβ and tau. However, although current glia-targeted strategies for AD remain inconclusive and mostly confined to preclinical studies, the high plasticity of glial cells and their crucial roles in the CNS make them highly promising therapeutic targets for future drug development.
